# Medical Education in Iraq: The cradle of Civilisation

**DOI:** 10.12669/pjms.35.3.972

**Published:** 2019

**Authors:** Shabih H. Zaidi, Ali Abutiheen

**Affiliations:** 1 *Shabih H. Zaidi, FRCS. London, UK*; 2 *Ali Abutiheen, University of Kerbala, Kerbala, Iraq*

**Keywords:** Medical Education, Arab World, Arab Health Boards

## Abstract

Medical education is in state of flux in Iraq. What used to be the best physicians and surgeons in the Arab world have fallen down to much lower status due to deliberate neglect and punishment of the intellectuals and professionals by the previous authorities. Once again medical educators and health professional are gaining momentum, gathering national and international support, to enter the contemporary world of medical education employing an integrated curriculum, PBL and Problem Solving Methods, Skill labs, simulation and structuring etc. Much needs to be done to return to formal glory but Iraqis are a dynamic nation of determined, dedicated and committed people. It is up to the international community to join hands with the Medics International to expedite the process. One such effort was the medical education conference held on 15-16 September 2018 with large input from medical educationists from Pakistan.

The ancient land of Mesopotamia located between the Tigris and the Euphrates, is aptly described as the cradle of civilisation. After all, the history here dates back to Adam and Noah, who are buried in Najaf. Prophet Ibrahim was born near the ancient Ur city; the capital of Sumerian empire, and where Prophet Dulkifal challenged the might of Nebuchadnezzar-2.

Long before the appearance of Akkadians, Assyrians, Sumerian, Aryans, the Indus valley civilization and others, this land of the Prophets and Saints introduced the fundamentals of civilisation to mankind. Hammurabi (c1750BC) ruled here introducing his famous code still present on a lead embedded clay tablet in the Louver in Paris. The remains of the Babylonian hanging gardens sit as a mount beside the gaily painted and never inhabited fort built by a later day dictator.

In September 2018, the University of Kerbala Medical School, engaged with Imamia Medics International^1^ to hold a 2-day conference in medical education, in the holy city of Karbala. This was the second instalment of the educational activity initiated in 2016. The first meeting was held between the faculty of Medical Education under Professor Turner of Edinburgh Medical School and the faculty of educators of Kerbala medical school. IMI’s Surgeon Ali Mehdi of Edinburgh, and Surgeon Ahsan Zaidi of London, in Hyderabad, India in February 2018, facilitated it. The Indian meeting created so much enthusiasm and excitement in the medical educators of Telangana State that according to the reliable sources; its competent authority has embarked upon engaging the faculty of medical education of Edinburgh for future collaboration.

The second instalment of that activity was held on 15-16 September 2018, which was an eye opener for many who travelled from various countries to participate. Earlier on, many top medical educators of Pakistan initiated a six month long exercise of bimonthly seminars and workshops in Lahore under the leadership of Professor Mulazim Hussain Bukhari from University of Lahore, Pakistan. They facilitated several workshops on the second day. The hosts had requested a series of workshops on contemporary topics as well as a seminar of plenary lectures by eminent teachers and scholars.

## DEVELOPMENTS IN PAKISTAN

Medical Education is enjoying an unprecedented popularity these days. More and more people are joining various certification courses to earn a diploma. Pakistan is mighty powerful in medical education. It started way back in late1970s and early 1980s when Professors Fazle Elahi and Naeem Jafry introduced the concept of medical education at the College of Physicians and Surgeons, Pakistan. SZ (the first author) was fortunate enough to attend the very first workshop facilitated by these great visionaries, where a WHO expert came to teach the Bloom’s Taxonomy and theories of teaching and learning. Prof Naeem Jafri used to say that a school teacher has to earn a diploma like BT or CT before joining the profession, while doctors have to do none. How true! Things have changed since.

## DEVELOPMENTS IN IRAN

Over the years, Pakistan has progressed more than any regional country except Iran which has an extraordinary programme of medical education in Tehran, Shiraz, Mash’hed and elsewhere. A visit to the Shiraz Medical Education Department in 2015, confirmed the amazing progress they have made. It is a pity that Iran faces isolation; otherwise we could learn so much from their centres, as well as their fully functional Virtual University.

September conference in Karbala coincided with the month of Muharram, so many participants gained not only knowledge but also spiritual satisfaction through pilgrimage to the shrines of Imam Ali, Imam Hussein o Hazrat Abbas and martyrs of Karbala. It was not only inspirational indeed motivational, to attend several workshops conducted by the facilitators. Details on some keynote presentations and workshop details are covered in Table-I.

**Table-I:** Scientific programme of medical education conference held in Kerbala.

*Building a career in research and academic medicine.*
Dr. Arshed Ali Quyyumi
Emory University School of Medicine, USA
***Ethics***: *The third dimension of medical education*
Dr. Shabih H. Zaidi
North London NHS Trust, UK
*Integrated curriculum in Kerbala*
Dr. Ali Abutiheen
College of Medicine, University of Kerbala, Iraq
*‘Publish or Perish’*
Dr. Fatema Jawad
Sindh Institute of Urology & Transplantation, Pakistan
*Medics International Virtual University*
Dr. Aamir Abbas
Khyber Medical University, Peshawar, Pakistan.
*Current & future trends in medical education in Iraq*
Dean Mohammad Saeed Abdulzahra
College of Medicine, University of Kufa, Iraq
*Complexity science and medical education*
Dr. Ali Tariq
College of Medicine, University of Kerbala, Iraq
*** Workshops & their Facilitators***
*Medical writing*
Dr. Fatema Jawad
SIUT, Karachi. Pakistan
*How to start a research project*
Dr. Aamir Abbas
Khyber Medical University, Peshawar, Pakistan
*Effective and timely feedback: Essential but often missing*
Dr. Tabassum Zehra
Aga Khan University Hospital, Karachi, Pakistan
*Implementation of reflective thinking in the University of Kufa*
Dr. Habib Al Hasani
College of Medicine, University of Kufa, Iraq
*Curriculum Development*
Dr. Shahzad Anwar
King Edward Medical University, Lahore, Pakistan
*Educational strategies & evaluation of curriculum*
Dr. Mulazim Hussain Bukhari
University of Lahore, Lahore, Pakistan
*Clinical governance: Its different parts and their application with examples from UK General Practice*
Dr. Imtiaz Gulamali
North London Family Practice, UK
*Peer Review*
Dr. Fatema Jawad
Sindh Institute of Urology & Transplantation, Karachi, Pakistan
*Biostats & introduction to SPSS*
Dr. Aamir Abbas
Khyber Medical University, Peshawar, Pakistan
*Clinical supervision of the run*
Dr. Tabassum Zehra
Aga Khan University Hospital, Karachi, Pakistan
*Applying Evidence Based Medicine in practice:*
*Particular example of how to do it.*
Dr. Imtiaz Gulamali
North London Family Practice, UK
*Twelve Tips for effective small group learning*
*Dr. Saima Batool*
University of Lahore, Lahore, Pakistan
*Integrated curriculum development*
– AKA Harden: An overview
Dr. Najmul Hassan Khan
Lahore General Hospital, Lahore, Pakistan
*Psychometrics, Assessment and Evaluation*
Dean Mohammad Saeed Abdulzahra
College of Medicine, University of Kufa, Iraq

An amazing plethora of information was disseminated during these two days. The inauguration attracted numerous Deans, Vice chancellors and top health officials from Baghdad, Mosul, Basra, Kufa, Qadisiyah, and Babylon universities. The discipline and etiquette observed during the function was exemplarily. The speeches were brief and to the point, and about 300 attendees engaged and fully engrossed.

### Healthcare in Iraq

It is a mixture of private – public partnerships. Many Iraqi doctors prefer to work in the state owned hospitals but also run their private practices, which are often quite lucrative. However, it has to be said that the condition of the public hospitals is not much better than what we have in some cities in the subcontinent. One major flaw appeared to be the lack of medical defence for any mistake, complications or negligence. It has thus driven many a surgeon opt out of surgery, leaving many an un operated goitre hanging grotesquely from the necks and hernias waiting to end up in complications. A senior physician said that the fear of revenge has driven many surgeons out of the country. To a question he replied that in case of a dispute the tribal chiefs settle the issue through a mutually agreeable compensation. The amount could be phenomenal, as the chief has to cater to the needs of his clan.

### Arab Health Boards

The Arab Board (arab-board.org) is the final certification body with its headquarters in Syria and regional offices in Jordan and Egypt. It covers nearly 20 countries. In a meeting with the high officials it was discussed that Pakistan and other nations could help in exchanging their faculties of master trainers, under graduate and postgraduate students and further training and transfer of technology. Even the Arab Board may be interested in inviting recognised examiners, as external examiners to assess the students and evaluate their programmes.

Historically, Iraq being an important component of colonial Britain had access to the best educational programmes akin to India. So in 1927, the first medical school was established in Baghdad by the British authorities, with Harry Sanderson, a seasoned physician as its dean.^2^ Of course this was the era, when Flexner’s traditional curriculum was implemented in all the British colonies as well as most US universities.

This curriculum was designed in an era when the world faced, calamities caused by major killers like Plague, Small pox, Syphilis, TB, Typhoid, Typhus, Cholera, and so forth. So Carnegie foundation, hired Abraham Flexner a non-medical educationist, to organise the teaching and training of medical students to combat prevailing clinical conditions. He based his curriculum on Biosciences, with the sole objective of producing a multitasking doctor. Social sciences including ethics were regrettably left out; thereby creating a huge vacuum in the practical application of the third dimension of the triangle of medical education formed by knowledge skills and attitude. Only now medical ethics is beginning to gain its due place.

Perhaps an answer could be to engage the ‘Hidden Curriculum’ more effectively to inculcate ethical practices in the lives of future students. Frederick Hafferty^3^,^4^ wrote in 1998 ‘all of what is taught during medical training is captured in course catalogues, class syllabi, lectures, notes and hand outs; indeed a great deal of what is taught- and most of what is learned –in medical school takes place not within formal course offerings but within medicine’s hidden curriculum’. ‘It is the set of influences that function at the level of organisational structure and culture including, for example, implicit rules to survive the institution such as customs, rituals, and taken for granted aspects’.^5^

Role modelling is an extremely potent component of hidden curriculum. ‘Role modelling takes place in different academic settings and is observed during formal, informal and hidden curriculum. Hidden curriculum operates and exerts its effects at the level of organizational system and practices. Hidden curriculum could be uncovered by the slangs used in the Institution e.g. using slang of business hub as if practice of medicine is a business’.^6^

Flexner’s curriculum has prospered in Iraq as well as numerous other countries since 1920s. Baghdad medical college taught and produced crops after crops of competent physicians and surgeons for over 80 years. One met several surgeons and physicians in later part of 1960s in London, who dominated the scene in the NHS. They were competent, capable and highly motivated doctors. They were the best amongst the Arab physicians.

Following Baghdad duly inspired by the Royal College of Surgeons of Edinburgh, Mosul, Basra, and Mustansirya Medical Colleges were established. The Kufa medical college came up in 1977,^7^ and the Kerbala Medical College in 2004 where PBL was introduced in 2013.^8^,^9^

Iraqi history is inundated with atrocities and calamities. So the Iraq-Iran conflict of 1980-88 imposed by previous regime on the Muslims of these countries destroyed all that Iraq possessed. Medical education was one of the casualties. 1990s saw huge economic sanctions, followed by the Bush-Blair war of 2003 that annihilated Iraq.

Previous regime had already forbidden the physicians from traveling abroad or outsiders to travel in to impart education. So after the glorious days of 1960s to 80s, the health services, medical education, teaching and training etc. collapsed. It is sad to listen to the stories of many seniors who could not attain their goals in life and now hope to catch up through international collaboration; some of them are Deans of Medical Colleges.

In 2016, there were 24 medical colleges in Iraq.^2^ In 2017, the first private college has opened. The curriculum is mostly traditional but since 2010, efforts have been made by many institutions to introduce an integrated curriculum based upon the principle of small group problem solving methods. It is integrated and student centred, but only Kerbala follows the PBL approach. The problem solving methods of Kufa is somewhat different. Unlike the traditional curriculum, the PBL is student driven and not teacher centred. The concept of gaining core knowledge during the medical school and more knowledge throughout life has caught on.

The Kufa Medical College under the able leadership of Dean Mohammad Saeed Abdul Zahra is one of the pioneers of clinically integrated curriculum. Baghdad and Kerbala have followed suit. Dean Riyadh Zubaidi of Kerbala took a bold step followed on by the current dean Dr Mayali. Babylon initiated the integrated curriculum this year, as did Duhok. Wasit joined this illustrious club four years ago.

The undergraduate programme is covered in six academic years. Following graduation and compulsory two years of rotational clerkship and a year of rural service, they can choose to specialise through the Iraqi or the Arab Board of certification. Usually the physicians require four years of training in medicine and five years in Surgery.^2^ However, it needs to be better structured and modernised. The authorities, especially the Arab Board, have initiated some efforts but more is needed.

It appears that each medical College chooses its own curriculum, instructional strategy and tools of assessment and evaluation. Furthermore, there is not enough or at least tailored or structured exposure of undergraduates to the clinical scenes. Postgraduates may be at the worst end. The training programme needs to improve, as postgraduates are skilled but not trained for attitude, communication skills, ethics and professionalism; essentially the 3^rd^ dimension of medical education. There are several reasons for this. One of them being the unattractive incentives for the supervisors, who are otherwise quite busy in their private practices; an ageless dilemma in many other developing countries!. What is worse is that the tribal culture and lack off indemnity, drive many a surgical trainee away from the profession, as was stated by a senior surgeon, who has opted out of surgical work to become a medical educator already deficient in number of physicians nationally.^10^ The health system and service delivery needs to be upgraded with medical education development with more efforts on primary health care services and increasing the number of trainee in Family Medicine.

## WAVE OF CHANGE IS SPREADING FAST

A wave of change is fast spreading elsewhere. In a subsequent meeting it was revealed that a national Board of Medical Education is already looking at various options to engage international academic institutions to bring Iraqi Medical education and health services compatible with international standards but duly emphasising on the fundamental target of meeting the local needs. Recently the British Council facilitated a meeting in Erbil, in which seven *Iraqi Medical* Schools namely Baghdad, Hawler, Kerbala, Kufa, Mustansiriya, Thi-Qar and Wasit. Their deans, professors, and policy makers met with Professor Nigel Bax and Professor Deborah Bax from the University of Sheffield. They discussed undergraduate education and how it could be developed to help recently qualified medical graduates deliver safe and effective care for patients.^11^

In several meetings held with the senior faculty, deans, health officials, executive of ministry of Higher Education and CEOS of various institutions, an urgent need was felt to assist in matters of both undergraduate and postgraduate education, curriculum development, teaching, training, assessment and evaluation. The proposal for the competent authority to consider are given in Table-II and Table-III.

**Table-II:** Suggestions to improve Medical Education.

1. A national task force may be formed to study the needs of the community today and in the foreseeable future.
2. To establish departments of medical education in all medical colleges. To consider to implement a clinically integrated curriculum uniformly at the national level.
3. To organise regular training of the trainers as Faculty Enhancement Programme.,
4. To develop close link ups with international institutions (like Kufa has done with Leicester and Kerbala is in consultation with Edinburgh).
5. Online courses through virtual learning platforms, universities, e-learning, engagement of contemporary modes of education as Moodle, MOOC, etc, robotics, skill labs, Simulated patients, professional actors, structured patients, artificial intelligence etc. Medics International Virtual University (MIVU) could act as a bridge with various international institutions.
6. Research and development are vital but lack badly. International collaboration could help. Emory, Einstein/Montefiore, Mount Sinai, Columbia, South Western, King’s in London and may others can be approached through the IMI’s academic council.
7. Although there are universities and the Iraqi Board for Medical Specialization as well as the Arab Board for Health Specialization, but they need to upgrade their curricula, training, assessment and evaluation process.
8. Focus on ethics, communication skills, attitude, professionalism, research methodology, writing and publishing is a matter of urgency. “Medical Education Council Iraq MECIQ” was recently founded, on initiation by the Arab Board with Dr. Hilal Al-Safar, a prominent speaker at the conference, as its head.

**Table-III:** Suggestions for the Health Board.

1. Grant fellowship, membership or postgraduate diplomas in various disciplines.
2. Organise structured postgraduate education nationwide.
3. Establish a department of Medical Education. Teaching and training of the supervisors and master trainers, development of Question bank, etc..
4. Design instructional strategies to match the needs of the syllabus based upon community needs.
5. Develop appropriate tools of assessment of the students and evaluation of the programme.
6. Engage with international institutions for exchange of students and faculty.
7. Conduct regular educational workshops, meetings, conferences etc.

In addition major institutions in Pakistan like the Aga Khan University have already established contacts with the emerging institutions in Iraq. The College of Physicians and Surgeons Pakistan and established universities the Punjab, like University of Health Sciences (UHS) and the Dow University of Health Sciences (DUHS) amongst others could play a major role in the whole process of training the trainers, faculty enhancement programmes capacity building and transfer of technology. Besides, many institutions in Iraq are willing to invite surgeons and specialist to serve the patients. Many Indian hospitals are already engaged in providing first grade services in special fields like Cardiac and Orthopaedic surgery. There is ample opportunity for Pakistan to send its teams of health experts to do the same for which the good offices of Imamia Medics International can be used to establish collaboration and cooperation in different fields.
